# Stroke Kinematics, Temporal Patterns, Neuromuscular Activity, Pacing and Kinetics in Elite Breaststroke Swimming: A Systematic Review

**DOI:** 10.1186/s40798-022-00467-2

**Published:** 2022-06-08

**Authors:** Emily Nicol, Simon Pearson, David Saxby, Clare Minahan, Elaine Tor

**Affiliations:** 1 Griffith Sports Science, Griffith University, Gold Coast, QLD Australia; 2 Queensland Academy of Sport, Brisbane, QLD Australia; 3 Griffith Centre of Biomedical and Rehabilitation Engineering, Griffith University, Gold Coast, QLD Australia; 4 Forethought, Melbourne, VIC Australia

**Keywords:** Swimming, Breaststroke, Biomechanics, Performance

## Abstract

**Background:**

Breaststroke is a technically complex stroke characterised by discontinuous propulsive phases, large intracyclic velocity variation and low mean velocity. The performance of this stroke at an elite level is influenced by a number of biomechanical, physiological and psychological factors. The present systematic review aimed to synthesise the biomechanical factors influencing elite breaststroke swimming performance. This review aims to provide elite coaches and performance scientists with a breadth of knowledge from which training and racing interventions can be developed.

**Methods:**

Electronic searches of Medline, Scopus and SPORTDiscus databases were conducted in May 2020 and March 2022. Search results that were peer-reviewed, published in English and published during or after the year 2000 were considered for review. The methodological rigour of studies was assessed using a risk of bias scale previously used for the evaluation of sports science research.

**Results:**

Thirty-eight articles were included in the present review. Articles investigated elite breaststroke performance in relation to one of the following areas: stroke kinematics, temporal patterns, neuromuscular activity, pacing and kinetics.

**Discussion:**

Kinematic, temporal and neuromuscular activity comparisons between groups of various race distance, performance or experience level, and athlete sex were common in the literature. These analyses demonstrated differences in stroke rate, stroke length, propulsive time, recovery time, glide time, sum of total integrated EMG and triceps brachii activation patterns between groups. The evaluation of various pacing strategies, and the relationship between kinetics and breaststroke performance was comparatively rare within the literature. Further research into the relationship between kinetics and breaststroke performance, and the manipulation of pacing strategy would increase the breadth of knowledge from which coaches and performance scientists can develop evidence-based training and racing interventions.

## Key Points

- Stroke kinematics (stroke rate, stroke length) and temporal patterns vary between 100 and 200 m events, and between male and female athletes. The 100 m event is typically characterised by higher stroke rate, lower stroke length, increased time spent in propulsive phases and a reduction in time spent in glide phases when compared to the 200 m event. Male swimmers typically have a higher stroke length, spend longer in propulsive phases and less time in the arm glide phase when compared to female swimmers of the same experience level.
- Use of small samples and the infrequency of studies that have investigated neuromuscular patterns, pacing strategies and kinetics in elite breaststroke populations limit the generalisability of existing findings. Further research in these areas is required to support current understanding.

## Background

Breaststroke is one of four competitive strokes contested at international swimming events. At the Olympic Games, breaststroke is raced over 100 m and 200 m distances, whilst an additional 50 m event is contested at World Championships. Breaststroke swimming is constrained by several rules that outline permitted technique. As defined by the swimming governing body, Federation Internationale De Natation (FINA):

1. After the start and after each turn, the swimmer may take one arm stroke completely back to the legs during which the swimmer may be submerged.
2. From the beginning of the first arm stroke after the start and after each turn, the body shall be on the breast. From the start and throughout the race, the stroke cycle must be one arm stroke and one leg kick in that order. All movements of the arms shall be simultaneous and on the same horizontal plane without alternating movement.
3. The hands shall be pushed forward together from the breast on, under or over the water. The elbows shall be under water except for the final stroke before the turn, during the turn and for the final stroke at the finish. The hands shall not be brought back beyond the hip line, except during the first stroke after the start and each turn.
4. During each complete cycle, some part of the swimmer’s head must break the surface of the water. All movements of the legs shall be simultaneous and on the same horizontal plane without alternating movement.
5. The feet must be turned outwards during the propulsive part of the kick.
6. At each turn and at the finish of the race, the touch shall be made with both hands separated and simultaneously at, above or below the water level. [1]

Technical rules result in several technique characteristics unique to breaststroke swimming. Dissimilar to other competitive strokes (backstroke, butterfly and freestyle) breaststroke is characterised by two discontinuous propulsive phases [2] and high resistive drag forces that result from underwater limb recoveries [3]. Due to these characteristics, breaststroke swimming produces the lowest mean velocity and the highest level of intracyclic velocity variation among the competitive strokes [4].

Despite the technical constraints placed on athletes during breaststroke events, a level of variability based on temporal characteristics, coordination patterns, neuromuscular activity and pacing profiles is still possible between individuals. In addition to producing variability between athletes, each of these parameters is suggested to influence breaststroke swimming performance at an elite level. The multiplicity of parameters reported to influence breaststroke swimming performance makes the identification of optimal training and racing strategies difficult.

At present no review has been performed on the biomechanics of elite breaststroke swimming. The present review aims to address this gap within the literature to synthesise the biomechanical factors influencing elite breaststroke swimming performance. Findings of this review will be of benefit to elite coaches and performance scientists in the development of training and racing interventions aimed at improving breaststroke swimming performance.

## Methods

### Search Strategy

Guidelines provided by the Preferred Reporting Items for Systematic Reviews and Meta-Analyses (PRISMA) were followed in this review [5]. A literature search was conducted in May 2020 across three electronic databases: Medline, Scopus and SPORTDiscus. Search filters were used to confine results to peer-reviewed articles published in English and published during or after the year 2000. Filters were used to ensure only recently published articles from trusted sources were considered for review. A combination of the following search terms was used: “breaststroke,” “biomechanics,” “technique,” “style,” “elite,” “national,” “international,” “anthropometry,” “flexibility” and “strength.”

### Selection Criteria

**Fig.1:**
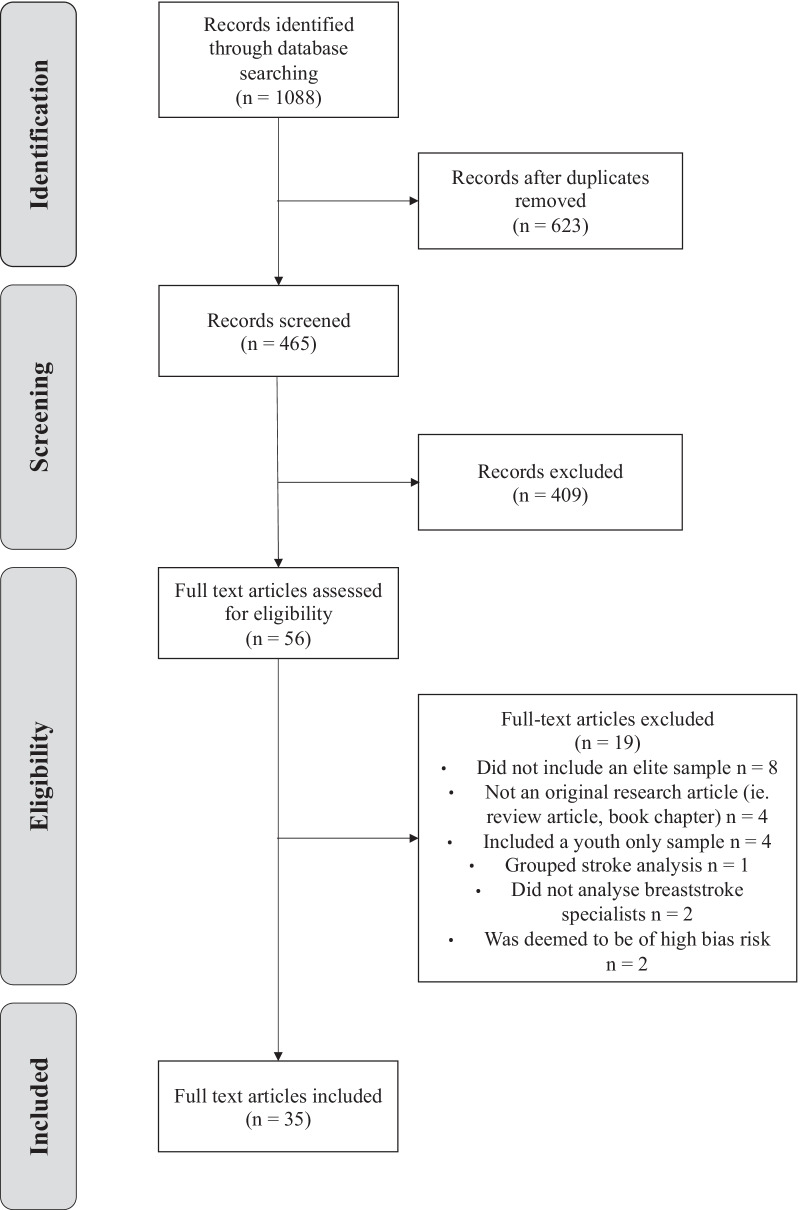
Search screening process following PRISMA guidelines

All search results were evaluated for eligibility using a number of criterion measures. Articles were excluded if (1) a full-text copy was unavailable, (2) the article was not original research, i.e. a review article, (3) the study was not conducted in a swimming pool environment, (4) breaststroke swimming was not investigated, (5) a non-elite or non-breaststroke sample was used, (6) a youth sample was used or (7) biomechanics was not a primary area of investigation. Figure 1 illustrates the search screening process.

### Quality Assessment

The quality of eligible studies was assessed using the risk of bias scale developed by Hindle et al. [6]. This scale is based on other evaluation checklists and has previously been used for the assessment of sports research [7, 8]. Sixteen standards were used to evaluate article quality: three standards to study design, four standards to sample characteristics, four standards to methodology and five standards to results and discussion. A detailed outline of assessment criteria is provided in Table 1. One point was awarded for each standard met to a maximum total of 16 points. No half points were awarded. Risk of bias score was subsequently determined using the total number of points awarded. Articles scored ≥ 11 points were categorised low bias risk. Articles scored 6–10 points were categorised satisfactory bias risk. Scores of < 5 were categorised high bias risk. Only articles with a low or satisfactory bias risk were included in the present review.

**Table 1:** Quality assessment scale

Element	Standard	Description
Study design	1.1	The study design is clearly stated
	1.2	The objectives/purpose of the study is clearly defined
	1.3	The design of the study adequately tests the hypothesis
Sample characteristics	2.1	The criteria for the inclusion of participants are clearly described
	2.2	The characteristics of the population is clearly described
	2.3	The study sample is representative of the population intended to the study
	2.4	A description of how the study size was arrived at is provided
Methodology	3.1	The testing methods are clearly described
	3.2	The measurement tools used are valid and reliable
	3.3	The statistical methods used well described
	3.4	The statistical tests used to analyse the data are appropriate
Results and discussion	4.1	The results are well described
	4.2	The information provided in the paper is sufficient to allow a reader to make an unbiased assessment of the findings of the study
	4.3	Confounding factors are identified
	4.4	Sponsorships/conflicts of interest are acknowledged
	4.5	Any limitations to the study are identified

## Results

### Study Characteristics

Following screening procedures and quality assessment, 38 articles were retained for review. Table 2 outlines publication details of articles contained within the present review. Of the 38 articles retained, 19 were categorised low bias risk and 19 were categorised as satisfactory bias risk (Table 3).

**Table 2:** Publication details of reviewed articles

Study	Author/s	Publication Year	Country	Journal
The influence of stroke mechanics into energy cost of elite swimmers	Barbosa et al. [31]	2008	Portugal	European Journal of Applied Physiology
Evaluation of arm-leg coordination in flat breaststroke	Chollet et al. [32]	2004	France	International Journal of Sports Medicine
Observation and technical characterisation in swimming: 200 m breaststroke	Conceição et al. [38]	2013	Portugal	Locomotor Apparatus in Exercise and Sports
Neuromuscular fatigue during 200 m breaststroke	Conceição et al. [24]	2014	Portugal	Journal of Sports Science and Medicine
Neuromuscular and motor patterns in breaststroke technique	Conceição et al. [40]	2019	Portugal	Brazilian Journal of Kineanthropometry & Human Performance
Analysis of speed, stroke rate, an stroke distance for world-class breaststroke swimming	Garland Fritzdorf et al. [30]	2009	Denmark	Journal of Sports Sciences
Differences between elite and sub-elite swimmers in a 100 m breaststroke: a new race analysis approach with time-series velocity data	Gonjo and Olstad [43]	2021	Norway	Sports Biomechanics
Difference muscle recruitment strategies among elite breaststrokers	Guignard et al. [11]	2015	France	International Journal of Sports Physiology and Performance
Kinematic measures and stroke rate variability in elite female 200 m swimmers in the four swimming techniques: Athens 2004 Olympic semi-finalists and French national 2004 championship semi-finalists	Hellard et al. [16]	2008	France	Journal of Sports Sciences
Relationships between swimming style and dry-land strength in breaststroke	Invernizzi et al. [21]	2014	Italy	Sports Sciences for Health
Do qualitative changes in interlimb coordination lead to effectiveness of aquatic locomotion rather than efficiency?	Komar et al. [26]	2014	France	Journal of Applied Biomechanics
Arm-leg coordination in flat breaststroke: a comparative study between elite and non-elite swimmers	Leblanc et al. [29]	2005	France	International Journal of Sports Medicine
Intracyclic distance per stroke phase, velocity fluctuations and acceleration time ratio of a breaststroker's hip: a comparison between elite and non-elite swimmers at different race paces	Leblanc et al. [3]	2007	France	International Journal of Sports Medicine
Stability of behaviour patterns in the 200 m breaststroke	Louro et al. [34]	2016	Portugal	Brazilian Journal of Kineanthropometry & Human Performance
Relationship between tethered forces and the four swimming technique performances	Morouço et al. [45]	2011	Portugal	Journal of Applied Biomechanics
The temporal analysis of elite breaststroke swimming during competition	Nicol et al. [33]	2021	Australia	Sports Biomechanics
Muscle activation in world-champion, world-class and national breaststroke swimmers	Olstad et al. [35]	2017a	Norway	International Journal of Sports Physiology and Performance
Muscular coordination, activation and kinematics of world-class and elite breaststroke swimmers during submaximal and maximal efforts	Olstad et al. [13]	2017b	Norway	Journal of Sports Sciences
Key factors related to short course 100 m breaststroke performance	Olstad et al. [15]	2020	Norway	International Journal of Environmental Research and Public Health
Changes in kinematics and arm-leg coordination during a 100 m breaststroke swim	Oxford et al. [14]	2017	UK	Journal of Sports Sciences
Analysis of selected kinematic and physiological performance determinants during incremental testing in elite swimmers	Psycharakis et al. [22]	2008	UK	Journal of Strength and Conditioning Research
Analysis of lap times in international swimming competitions	Robertson et al. [44]	2009	Australia	Journal of Sports Sciences
An approach to identifying the effect of asymmetries on body alignment in swimming exemplified by a case study of a breaststroke swimmer	Sanders et al. [47]	2015	Australia	Journal of Sports Science and Medicine
A new index of flat breaststroke propulsion: a comparison of elite men and women	Seifert and Chollet [18]	2005	France	Journal of Sports Sciences
Modelling spatial–temporal and coordinative parameters in swimming	Seifert and Chollet [36]	2009	France	Journal of Science and Medicine in Sport
Interlimb coordination and energy cost in swimming	Seifert et al. [37]	2013	France	Journal of Science and Medicine in Sport
Coordination pattern adaptability: energy cost of degenerate behaviours	Seifert et al. [27]	2014	France	PLoS One
Reproducibility of pacing profiles in elite swimmers	Skorski et al. [41]	2014	Germany	International Journal of Sports Physiology and Performance
Accelerometer profile of motion of the pelvic girdle in breaststroke swimming	Staniak et al. [28]	2016	Poland	Journal of Human Kinetics
Differences in stroke phases, arm-leg coordination and velocity fluctuation due to event, gender and performance level in breaststroke	Takagi et al. [2]	2004	Japan	Sports Biomechanics
An analysis of selected kinematic variables in national and elite male and female 100 m and 200 m breaststroke swimmers	Thompson et al. [12]	2000	UK	Journal of Sports Sciences
The effect of even, positive and negative pacing on metabolic, kinematic and temporal variables during breaststroke swimming	Thompson et al. [42]	2003	UK	European Journal of Applied Physiology
A comparison of selected kinematic variables between races in national and elite male 200 m breaststroke swimmers	Thompson et al. [23]	2004	UK	Journal of Swimming Research
The effects of changing pace on metabolism and stroke characteristics during high-speed breaststroke swimming	Thompson et al. [17]	2004	UK	Journal of Sports Sciences
Use of pressure distribution analysis to estimate fluid forces around a foot during breaststroke kicking	Tsunokawa et al. [46]	2015	Japan	Sports Engineering
Muscle coordination during breaststroke swimming: comparison between elite swimmers and beginners	Vaz et al. [39]	2016	Portugal	Journal of Sports Sciences
Sex-related differences and age of peak performance in breaststroke versus freestyle swimming	Wolfrum et al. [19]	2013	Switzerland	BMC Sports Science, Medicine and Rehabilitation
Changes in breaststroke swimming performances in national and international athletes competing between 1994 and 2011: a comparison with swimming performances	Wolfrum et al. [20]	2014	Switzerland	BMC Sports Science, Medicine and Rehabilitation

**Table 3:** Quality assessment of reviewed and excluded articles

Study	Publication Year	1.1	1.2	1.3	2.1	2.2	2.3	2.4	3.1	3.2	3.3	3.4	4.1	4.2	4.3	4.4	4.5	Total
Barbosa et al. [31]	2008	*	*			*			*	*	*	*	*	*				9
Chollet et al. [32]	2004	*	*			*			*		*	*	*	*				8
Conceição et al. [38]	2013	*	*			*	*		*		*	*	*	*		*		10
Conceição et al. [24]	2014	*	*	*		*			*		*	*	*	*				9
Conceição et al. [40]	2019	*	*			*			*				*	*		*		7
Garland Fritzdorf et al. [30]	2009	*	*		*		*						*				*	6
Gonjo and Olstad [43]	2021	*	*	*		*			*	*	*	*	*	*	*	*	*	13
Guignard et al. [11]	2015	*	*			*				*	*	*		*				7
Hellard et al. [16]	2008	*	*	*	*	*	*		*	*	*	*	*	*		*		13
Invernizzi et al. [21]	2014	*	*			*	*		*	*	*	*	*	*	*	*		12
Komar et al. [26]	2014	*	*	*		*			*	*	*	*	*	*		*	*	12
Leblanc et al. [29]	2005	*	*			*			*		*	*	*	*				8
Leblanc et al. [3]	2007	*	*	*					*	*	*	*	*	*				9
Louro et al. [34]	2016	*	*	*		*			*	*							*	7
Morouço et al. [45]	2011	*	*		*	*			*		*	*	*	*	*			10
Nicol et al. [33]	2021	*	*	*		*	*		*	*	*	*	*	*		*		12
Olstad et al. [35]	2017	*	*	*		*			*	*	*	*	*	*			*	11
Olstad et al. [13]	2017	*	*			*			*	*	*	*	*	*		*	*	11
Olstad et al. [15]	2020	*	*	*		*	*		*	*	*	*	*	*		*	*	13
Oxford et al. [14]	2017	*	*	*	*	*			*	*	*	*	*	*	*	*		13
Psycharakis et al. [22]	2008	*	*		*	*		*	*	*	*	*	*	*		*	*	13
Robertson et al. [44]	2009	*	*				*		*	*	*	*	*	*	*		*	11
Sanders et al. [47]	2015	*	*		*	*		*	*	*	*	*	*	*				11
Seifert and Chollet [18]	2005	*	*	*		*			*		*	*	*	*				9
Seifert and Chollet [36]	2009	*	*			*	*		*	*	*	*	*	*	*			11
Seifert et al. [27]	2014	*	*	*		*			*	*	*	*	*	*		*		11
Seifert et al. [37]	2013	*	*	*		*			*	*	*	*	*	*		*		11
Skorski et al. [41]	2014	*	*	*	*	*	*	*	*	*	*	*	*	*	*		*	15
Staniak et al. [28]	2016	*	*			*			*	*	*	*	*	*		*		10
Takagi et al. [2]	2004	*	*		*			*	*		*	*	*	*	*			10
Thompson et al. [12]	2000	*	*				*		*		*	*	*	*				8
Thompson et al. [42]	2003	*	*			*			*	*	*	*	*	*			*	10
Thompson et al. [23]	2004	*	*		*		*		*		*	*	*	*			*	10
Thompson et al. [17]	2004	*	*			*			*		*	*	*	*				8
Tsunokawa et al. [46]	2015	*	*			*			*	*	*		*	*		*	*	10
Vaz et al. [39]	2016	*	*	*		*			*		*	*	*	*	*	*	*	12
Ward [NA]	2018	*	*		*						*	*						5
Wolfrum et al. [19]	2013	*	*	*			*	*	*	*	*	*	*	*	*	*	*	14
Wolfrum et al. [20]	2014	*	*	*	*		*	*	*	*	*	*	*	*	*	*	*	15
Xin-Feng et al. [NA]	2007	*	*						*				*	*				5

*Refer to Table 1 for criterion definitions

The most commonly used method for data collection in the eligible articles was videography (*n* = 15). An additional six studies used a combination of electromyography (EMG) and videography, six studies analysed retrospective race data and four studies used hand timing or pacing technology throughout data collection. Other data collection methods included the use of a linear position transducer and videography (*n* = 3), accelerometry (*n* = 1), force gauge (*n* = 1), pressure sensors (*n* = 1) and EMG without videography (*n* = 1).

### Videography

A total of 24 studies used videography throughout data collection. Fifteen of these studies used videography as the sole method of data collection. Videography-based studies used between one and 11 cameras during data collection. Table 4 provides further details regarding the methodology used and themes discussed throughout each of the 15 exclusively videography-based studies. The majority of these studies analysed breaststroke swimming in two-dimensions (2D) (*n* = 12). All studies that conducted 2D analysis of breaststroke swimming investigated temporal and kinematics characteristics of breaststroke swimming within an elite population. Group comparisons based on race distance, experience level and sex were frequently discussed within 2D videography studies.

**Table 4:** Outline of videography studies

Study	Publication year	Themes	Number of participants	Speed of swimming	Number of cameras used	Dimensionality of analysis	Parameters measured
Gonjo and Olstad [43]	2021	Kinematics Experience-level comparison	7 elite male swimmers 7 sub-elite male swimmers	Time trial	10	2D	Velocity Race segment analysis
Hellard et al. [16]	2008	Kinematics Experience-level comparison	16 female international-level semi-finalists 16 female national-level semi-finalists	In competition	4	2D	Stroke rate Stroke length Velocity
Invernizzi et al. [21]	2014	Strength expression	24 male national-level swimmers 20 female national-level swimmers	Time trial	1	2D	Stroke rate Stroke length Velocity Normalised chin-up score Normalised jump-reach score
Komar et al. [26]	2014	Temporal analysis Experience-level comparison	5 male expert swimmers 3 female expert swimmers 6 male recreational swimmers 4 female recreational swimmers	Race pace simulation	6	3D	Velocity Intracyclic velocity variation Displacement Acceleration Elbow angle Knee angle
Louro et al. [34]	2016	Temporal analysis Individual analysis	5 male national-level swimmers	Time trial	2	2D	Movement events Stroke phases
Olstad et al. [15]	2020	Kinematics	15 male high-level swimmers	Time trial	11	2D	Velocity Race segments analysis Stroke rate Stroke length Glide distance Stroke count
Oxford et al. [14]	2017	Kinematics Temporal analysis	18 male national-level swimmers 8 female national-level swimmers	Time trial	3	2D	Stroke rate Stroke length Velocity La^+^ Heart rate RPE Stroke phases
Sanders et al. [47]	2015	Kinematics Asymmetry	1 elite female swimmer	Fatigue set	6	3D	Displacement Acceleration Angular velocity Peak torque
Seifert and Chollet [18]	2005	Temporal analysis Race distance comparison Sex comparison	9 elite male swimmers 8 elite female swimmers	Race pace simulation	3	2D	Stroke rate Stroke length Velocity Index of flat breaststroke propulsion Stroke phases
Seifert and Chollet [36]	2009	Temporal analysis Race distance comparison	12 elite male swimmers	Race pace simulation	4	2D	Stroke rate Stroke length Velocity Stroke phases
Seifert et al. [37]	2013	Coordination pattern manipulation Energy cost	8 male national-level swimmers	Submaximal	2	2D	Stroke rate Stroke length VO_2_ La^+^
Seifert et al. [27]	2014	Coordination pattern manipulation Energy cost	7 national-level swimmers ^a^	Submaximal	6	3D	VO_2_ La^+^ Energy cost Intracyclic velocity variation Angular velocity Trunk inclination Elbow angle Knee angle Stroke phases
Takagi et al. [2]	2004	Temporal analysis Race distance comparison Experience-level comparison	15 male 50 m international races 16 male 100 m international races 15 male 200 m international races 12 female 50 m international races 10 female 100 m international races 13 female 200 m international races	In competition	3	2D	Stroke rate Stroke length Velocity Intracyclic velocity variation Stroke phases
Thompson et al. [12]	2000	Kinematics Race distance comparison	159 male 100 m international- or national-level finals 158 female 100 m international- or national-level finals 159 male 200 m international- or national-level finals 158 female 200 m international- or national-level finals	In competition	5	2D	Stroke rate Stroke length Velocity Skill time
Thompson et al. [23]	2004	Kinematics Individual between race comparison	36 male international- or national-level finalists	In competition	5	2D	Stroke rate Stroke length Velocity Skill time

^a^Participant sex not specified

The remaining three videography studies analysed breaststroke swimming in three-dimensions (3D). The comparatively small number of studies that used 3D methodology may be attributed to the time-consuming and resource-demanding procedures required of this method [9, 10]. Each of these three 3D-based studies had different aims and procedures, but met these aims through the investigation of similar parameters (acceleration, displacement, angular velocity and joint angles) (Table 4).

### Electromyography Methods

Seven studies used EMG during data collection. All studies with the exception of Guignard et al. [11] combined EMG analysis with videography. EMG-based studies involved the fixation of bipolar surface electrodes (*n *= 4) or triode surface electrodes (*n* = 3) to the skin surface directly above various muscle groups for the measurement of neuromuscular activity. All EMG-based studies collected neuromuscular information wirelessly, and all sampled at 1000 Hz. EMG studies investigated the activation patterns of the following eight muscles: biceps brachii, pectoralis major, trapezius, triceps brachii, biceps femoris, gastrocnemius, rectus femoris and tibialis anterior. Table 5 details the methods used and themes discussed within each of the seven EMG-based studies. The analysis of neuromuscular activity was frequently combined with a kinematic analysis from videography. Group comparisons based on experience level were frequently made and discussed.

**Table 5:** Outline of EMG studies

Study	Publication year	Themes	Number of participants	Speed of swimming	Number of EMG sensors used	Location of EMG sensors	Number of cameras Used	Dimensionality of analysis	Parameters measured
Conceição et al. [38]	2013	Neuromuscular activity Kinematics	12 male national-level swimmers	Time trial	4	Biceps brachii Deltoid anterior Pectoralis major Triceps brachii	2	2D	Stroke rate Stroke length Velocity La^+^
Conceição et al. [24]	2014	Fatigue Neuromuscular activity Kinematics	9 male national-level swimmers	Time trial	4	Biceps brachii Deltoid anterior Pectoralis major Triceps brachii	2	2D	Stroke rate Stroke length Velocity La^+^ Stroke index
Conceição et al. [40]	2019	Neuromuscular activity Temporal patterns	5 male national-level swimmers	Time trial	4	Biceps brachii Deltoid anterior Pectoralis major Triceps brachii	2	2D	Temporal patterns Body undulation
Guignard et al. [11]	2015	Neuromuscular activity Individual analysis	1 female international-level swimmers 2 female national-level swimmers	Race pace simulation	4	Biceps femoris Gastrocnemius Rectus femoris Tibialis anterior	NA	NA	Knee angle Ankle angle Thigh angle Stroke phases
Olstad et al. [35]	2017	Neuromuscular activity Temporal analysis Experience-level comparison	2 world-class male swimmers 2 national-elite male swimmers 2 world-class female swimmers 2 national-elite female swimmers	Race pace simulation	8	Biceps brachii Pectoralis major Trapezius Triceps brachii Biceps femoris Gastrocnemius Rectus femoris Tibialis anterior	6	3D	Stroke rate Stroke length Velocity Knee angle Maximal voluntary contraction Stroke phases
Olstad et al. [13]	2017	Neuromuscular activity Kinematics Intensity differences	4 elite male swimmers 5 elite female swimmers	Race pace simulation	8	Biceps brachii Pectoralis major Trapezius Triceps brachii Biceps femoris Gastrocnemius Rectus femoris Tibialis anterior	16	3D	Stroke rate Stroke length Velocity Knee angle Maximal voluntary contraction Stroke phases
Vaz et al. [39]	2016	Neuromuscular activity Experience-level comparison	4 elite male swimmers 4 elite female swimmers 4 beginner male swimmers 4 beginner female swimmers	Race pace simulation	8	Biceps brachii Pectoralis major Trapezius Triceps brachii Biceps femoris Gastrocnemius Rectus femoris Tibialis anterior	6	2D	Knee angle Stroke phases

### Retrospective Race Data Methods

Six studies used retrospective competition data for the analysis of elite breaststroke swimming. This approach required the collation and analysis of existing competition splits, times, metadata and race footage. The number and level of analysed competitions are detailed in Table 6. Total race and split times were typically used to determine pacing profiles and race speed characteristics. Three studies used this information to make between-group comparisons based on sex, age and/or experience level. Two studies used pacing and speed data to compare individual results between competitions. The final study used retrospective race footage to calculate the amount of time spent in various stroke phases and determine temporal differences between groups based on race distance and sex.

**Table 6:** Outline of retrospective race data studies

Study	Publication year	Themes	Number of races analysed	Level and date range of competition	Parameters measured
Garland Fritzdorf et al. [30]	2009	Effective work per stroke Individual race comparison	14 male 100 m breaststroke races. 7 races of various world ranked swimmers and 7 races of a single world ranked swimmer	NA	Total race time Split time Effective work per stroke
Nicol et al. [33]	2021	Temporal analysis Race distance comparison Sex comparison	20 male 100 m national-level races 15 male 200 m national-level races 24 female 100 m national-level races 27 female 200 m national-level races	National and international-level competitions over a 3 year period	Stroke phase time Total race time
Robertson et al. [44]	2009	Pacing Stroke comparison Experience-level comparison	1530 male races ^a,b^ 1527 female races ^a,b^	9 international-level competitions over a 7 year period	Total race time Split time Race position
Skorski et al. [41]	2014	Pacing Individual race comparison	362 male races from 158 male athletes ^a^ 70 male 200 m breaststroke races	22 national and international-level competitions over a 1 year period	Total race time Split time Average velocity
Wolfrum et al. [19]	2013	Sex comparison Experience-level comparison Age group comparison Race speed	14,166 Swiss female races ^a,b^ 14,798 Swiss male races ^a,b^ 240 international-level female races ^a,b^ 240 international-level male races ^a,b^	Swiss athletes: national-level competition over a 4 year period International athletes: NA	Average swimming speed
Wolfrum et al. [20]	2014	Sex comparison Race speed	NA	Swiss athletes: best performances of the top 10 Swiss male and female athletes over a 17 year period International athletes: 8 international-level competitions over a 17 year period	Average swimming speed

^a^Multiple strokes analysed

^b^Number of breaststroke races analysed unspecified

### Other Analysis Methods

The following data collection methods were used by fewer than four studies within the dataset: linear position transducer with videography (*n* = 3), pacing lights (*n* = 3), force gauge (*n* = 1), hand timing (*n* = 1), accelerometers (*n* = 1) and pressure sensors (*n* = 1). Themes of discussion varied widely between these studies. Table 7 outlines the samples used and themes discussed within each of these studies.

**Table 7:** Outline of studies with unique methodology

Study	Publication year	Themes	Number of participants	Speed of swimming	Methodology used	Methodology details	Parameters measured
Barbosa et al. [31]	2008	Kinematics Energy Cost	3 international-level male swimmers 2 international-level female swimmers	Submaximal	Pacing lights		Stroke rate Stroke length Velocity VO_2_ La^+^ Energy cost Energy expenditure
Chollet et al. [32]	2004	Temporal analysis Race distance comparison	9 male expert swimmers 7 female expert swimmers	Race pace simulation	Linear position transducer & videography	3 cameras used for 2D videography analysis	Stroke rate Stroke length Velocity Stroke phases
Leblanc et al. [29]	2005	Temporal analysis Race distance comparison Experience-level comparison	11 national- and international-level male swimmers 9 national- and international-level female swimmers 11 regional-level male swimmers 9 regional-level female swimmers	Race pace simulation	Linear position transducer & videography	3 cameras used for 2D videography analysis	Stroke rate Stroke length Velocity Stroke phases
Leblanc et al. [3]	2007	Temporal analysis Kinematics Experience-level comparison	9 national-level male swimmers 9 regional-level male swimmers	Race pace simulation	Linear position transducer & videography	3 cameras used for 2D videography analysis	Stroke rate Stroke length Velocity Intracyclic velocity variation Acceleration-deceleration time ratio Stroke phases
Morouço et al. [45]	2011	Force Velocity	8 international-level female swimmers	Race pace simulation	Force gauge	Load cell attached to a steel cable and affixed to a belt worn around participants’ waist	Velocity Force Height Weight Hydrostatic mass Surface area
Psycharakis et al. [22]	2008	Kinematics Fatigue Physiology	2 international-level male swimmers 2 international-level female swimmers	Submaximal	Hand timing		Stroke rate Stroke length Velocity La^+^
Staniak et al. [28]	2016	Accelerometry Temporal analysis	5 elite male swimmers	Submaximal	Accelerometry	1 accelerometer positioned on dorsally on the pelvic girdle	Acceleration Angular velocity Stroke phases
Thompson et al. [17]	2004	Kinematics Physiology Pacing	9 national-level male swimmers	Time trial	Aquapacer™		Stroke rate Stroke count VO_2_ La^+^ Heart rate Rate of perceived exertion Height Weight Skinfolds Hydrostatic mass
Thompson et al. [42]	2003	Kinematics Physiology Pacing	9 national-level male swimmers	Time trial	Aquapacer™		Stroke rate Stroke count VO_2_ La^+^ Heart rate Rate of perceived exertion Height Weight Skinfolds
Tsunokawa et al. [46]	2015	Fluid force Velocity	8 national-level male swimmers	Race pace simulation	Pressure sensors	8 sensors positioned on the foot	Force Fluid force Impulse

## Discussion

A multitude of factors have been reported to influence breaststroke technique and performance at an elite level. Within the existing literature, a number of themes are frequently discussed. These include stroke kinematics, temporal characteristics, neuromuscular activity, pacing and kinetics. The following sections will discuss each theme with reference to the existing literature.

### Stroke Kinematics

Average horizontal velocity, measured by the time to cover a given distance, is the primary outcome measure used to assess swimming competition performance. Kinematic parameters referenced within the swimming biomechanics literature are consequently described in relation to their influence on swimming velocity. Table 8 details the average velocity values reported within each reviewed study where available. Two of the most frequently referenced kinematic parameters in breaststroke swimming biomechanics are stroke rate (SR) and stroke length (SL).

**Table 8:** Stroke rate, stroke length and average velocity reported ranges and calculation methods

Study	Publication year	Swimming pace	SR Calculation (strokes per min)	Reported SR range	SL Calculation (m per stroke)	Reported SL range	Reported v (m/s)
Barbosa et al. [31]	2008	Submaximal	Stopwatch measure over three stroke cycles	NA	v/SR	NA	NA
Conceição et al. [38]	2013	200 m	^a^	Male: 34.40 ± 3.58–37.52	^a^	Male: 1.96 ± 0.24–2.32 ± 0.37	Male: 1.16 ± 0.09–1.41 ± 0.07
Conceição et al. [24]	2014	200 m	1/stroke cycle length	Male: 34.80 ± 2.83–37.58 ± 4.90	^a^	Male: 1.92 ± 0.15–2.23 ± 0.18	Male: 1.14 ± 0.08–1.38 ± 0.09
Hellard et al. [16]	2008	200 m	60/stroke duration	Male: 35.7 ± 3.1–37.9 ± 4.2	v/SR/60	Male: 1.94 ± 0.17–2.18 ± 0.26	Male: 1.18 ± 0.02–1.33 ± 0.02
Komar et al. [26]	2014	70% and 90% of maximal speed	NA	NA	^a^	Male and female: 1.81 ± 0.33–2.78 ± 0.31	Male and female: 1.08 ± 0.11–1.37 ± 0.10
Leblanc et al. [3]	2007	50 m, 100 m and 200 m	Stopwatch measure over three stroke cycles	Male: 39.22 ± 3.23–51.91 ± 5.21	^a^	Male: 1.80 ± 0.26–2.15 ± 0.18	Male: 1.40 ± 0.10–1.53 ± 0.12
Olstad et al. [13]	2017	60%, 80% and 100% of maximal speed	^a^	Male and female: 32.20 ± 3.43–42.58 ± 4.36	^a^	Male and female: 1.70 ± 0.17–1.90 ± 0.21	Male and female: 1.04 ± 0.13–1.20 ± 0.16
Olstad et al. [15]	2020	100 m	^a^	Male: 49.62 ± 4.04–53.28 ± 4.01	^a^	Male: 1.58 ± 0.13–1.71 ± 0.11	Male: 1.32 ± 0.06–1.51 ± 0.07
Oxford et al. [14]	2017	100 m	^a^	Male: 43.7 ± 5.6–46.8 ± 7.4 Female: 47.2 ± 8.4–49.7 ± 8.2	^a^	Male: 1.55 ± 0.24–1.64 ± 0.22 Female: 1.28 ± 0.22–1.39 ± 0.24	Male: 1.13 ± 0.07–1.24 ± 0.1 Female: 1.00 ± 0.08–1.11 ± 0.06
Psycharakis et al. [22]	2008	Submaximal	Stopwatch measure over three stroke cycles	NA	v/SR	NA	NA
Thompson et al. [12]	2000	100 m and 200 m	Number of frames taken to complete a single stroke cycle immediately following the 25 m mark	Male 100: 49.2 ± 5.4–51.0 ± 5.2 Female 100: 49.5 ± 5.8–49.7 ± 5.7 Male 200: 37.1 ± 4.5–43.0 ± 5.9 Female 200: 38.8 ± 5.3–43.4 ± 5.7	v/SR	Male 100: 1.67 ± 0.17–1.85 ± 0.30 Female 100: 1.52 ± 0.18–1.63 ± 0.19 Male 200: 1.84 ± 0.25–2.22 ± 0.25 Female 200: 1.66 ± 0.21–1.89 ± 0.25	Male 100: 1.40 ± 0.06–1.49 ± 0.05 Female 100: 1.24 ± 0.07–1.33 ± 0.07 Male 200: 1.31 ± 0.12–1.41 ± 0.07 Female 200: 1.18 ± 0.06–1.27 ± 0.07
Thompson et al. [23]	2004	200 m	^a^	Male: 37.03 ± 4.38–43.26 ± 4.28	v/SR	Male: 1.88 ± 0.19–2.28 ± 0.23	Male:1.34 ± 0.05–1.46 ± 0.05

^a^Calculation method unclear

Stroke kinematic characteristics including SR and SL vary by race distance and race duration. The 100 m event is characterised by higher mean SR and lower mean SL when compared to the 200 m event [2, 3, 12]. This pattern is consistent during various intensity efforts, with increases to SR and decreases to SL associated with increase in intensity [13]. Stroke kinematics have also been reported to change over the duration of an event; however, the reported direction of these changes is inconsistent. Whilst SR decreases over the duration of a 100 m event have been reported during short course (25 m pool) efforts [14, 15], SR increases over the duration of a 100 m event have been reported in long-course (50 m pool) efforts [12]. Discrepancy in the reported direction of SR and SL changes over a 100 m event may be attributed to variance in the calculation of SR between studies (Table 8) or to a difference in race profiles between short course and long-course events. An increase in SR across race duration is also reported to occur during the long-course 200 m event when comparing first and second 100 m sections [12, 16, 17]. An increase in SR over the latter part of a long-course 100 m or 200 m event is suggested to be a compensatory strategy for SL reduction [12, 17]. Reduction in velocity over the final 50 m of a 100 m event irrespective of an increase in SR suggests that SR increases are not sufficient to overcome the effects of decreased SL [12].

Stroke kinematics also vary according to a number of fixed and modifiable athlete characteristics. One such characteristic is the sex of the athlete. Male swimmers typically have a longer stroke length than female swimmers at 100 m and 200 m race distances [2, 14]. This sex-related difference is attributed to the greater height of male swimmers when compared to female swimmers [18]. Elite male swimmers also maintain a higher average velocity than female swimmers across all race distances [14, 19, 20]. The magnitude of sex-related velocity differences, however, decreases with increase in race distance [19, 20]. This observation has been attributed to a greater swimming efficiency in female swimmers when compared to male swimmers [20]. Meaningful sex-related differences in SR are yet to be established. A modifiable athlete characteristic, muscular strength, is also said to influence stroke kinematics. Invernizzi et al. [21] reported swimmers who achieved a high countermovement movement jump score adopted a stroke with high SL. Conversely, swimmers who scored highly on an exhaustive chin-up test adopted a stroke with higher SR. Results from Invernizzi et al. [21] suggest individuals adopt a SR to SL ratio based on their strength attributes. This suggestion is consistent with much of the existing literature that suggests optimal SR to SL ratios are best determined on an individual basis [12, 15, 22, 23] with consideration of athlete anthropometry, technique, flexibility and coordination [22]. The individualised nature of optimal SR to SL ratios may also explain the weak and inconsistent relationships between these kinematic parameters and swimming velocity in cross-sectional group analyses.

As well as their use as descriptive measures to assess breaststroke swimming, SR and SL have also been used to assess stroke efficiency. Defined as an athlete’s ability to travel at a specified velocity with the fewest number of strokes, breaststroke efficiency may be assessed using stroke index (SI) (Eq. 1) [24, 25]

1 $${\text{SI}} = {\text{average }}\;{\text{velocity}}*{\text{SL}}$$

Higher values of SI indicate greater swimming efficiency. This measure of efficiency assumes that the swimmer with the greatest stroke length at a given velocity has the best efficiency [25]. As it is understood that optimal SR and SL ratios exist for individuals, stroke index may consequently be better used to assess intraindividual efficiency patterns rather than as a method of efficiency comparison between athletes. Despite this assumption, common patterns in SI based on sex and race distance have been identified. Male swimmers typically have a higher SI when compared to female swimmers [14]. According to the SI model, this finding would suggest that male swimmers swim with greater efficiency than female swimmers. The higher SI in male swimmers may instead reflect male-specific velocity and SL patterns rather than superior efficiency given that female swimmers have been reported to maintain higher swimming efficiency due to body morphology differences [20]. This highlights a delimitation of SI as a method of intra-athlete efficiency comparison.

Over the course of a 200 m event, stroke efficiency (assessed using SI) has been reported to decrease (3.07 ± 0.25–2.19 ± 0.29 m^2^/s) [24]. The reduction in SI reflects reported kinematic changes to SL and swimming velocity that occur as race duration increases.

Another method used to assess breaststroke efficiency is intracyclic velocity variation (IVV). Quantified using time–velocity information IVV is calculated using Eq. 2 [26, 27].

2 $${\text{IVV}} = \frac{{\left( {{\text{Max}}\;L - {\text{Min}}\;L} \right) + \left( {{\text{Max}}\;A - {\text{Min}}\;T} \right)}}{V}$$

Where MaxL corresponds to the maximum velocity achieved during the leg propulsion phase, MinL corresponds to the minimum velocity achieved during maximal knee flexion, MaxA corresponds to the maximum velocity achieved during the arm propulsive phase, and MinT corresponds to the minimum velocity achieved during body glide [26, 27]. Variations of this formula have been used by various research groups for similar stroke assessment [3]

Large IVV values are considered disadvantageous to swimming performance [28]. This is due to a consequent need to overcome higher inertial forces following large decelerations in order to initiate acceleration throughout propulsive phases [27, 29]. IVV may be reduced through a shorter glide time and a consequent reduction in the amount of time spent in a deceleration phase [27]. This reduction in glide time may explain the reported reduction in IVV at higher swimming speeds [26]. Leblanc et al. [3] challenged this idea, finding that IVV did not vary between race paces. Contradictory findings may be attributed to the use of different data collection methods. The calculation of velocity using centre of mass displacement [26] rather than the use of hip displacement as measured using a linear position transducer [3] limits the extreme values of calculated velocity [26]. This may account for lower levels of IVV reported in 3D video-based studies when compared to linear position transducer-based studies.

Effective work per stroke (eWPS) is an additional method that has been used to calculate and assess stroke effectiveness in breaststroke swimming (Eq. 3) [30].

3 $${\text{eWPS}} \left( \% \right) = 100\left( {\frac{{V_{i} - V_{m} }}{{V_{m} }}} \right)$$

Where *V*_*i*_ are achieved speed values and *V*_*m*_ are modelled or expected speed values [30]. Although eWPS has been associated with changes in mean race speed, and flatter effectiveness profiles with faster overall race time, this method of analysis is limited due to its assumption that effectiveness remains stable as SR changes [30]. It is also limited in its assumption that drag levels experienced by an individual remain constant across various speeds [30]. Effective work per stroke is consequently infrequently reported within the literature.

Stroke kinematics have also been associated with physiological cost. Changes to both SR and SL are associated with changes in energetics in breaststroke swimming [31]. Increases in SR are associated with an increase in energy cost (*R*^2^ = 0.17, *p* < 0.05) [31]. Conversely, increases in SL are associated with decreased energy cost (*R*^2^ = 0.24, *p* < 0.05) [31]. Despite weakness in the associations between SR, SL and energy cost, changes to SR and SL account for 53% and 40% of variance in energy cost, respectively [31].

### Temporal Analysis

Temporal analysis was often discussed with reference to stroke phases and/or coordination patterns. The stroke cycle was commonly described in two broad phases: pull and kick. Within each phase, a number of subphases were described based on observable movement patterns. Table 9 outlines various stroke phase models used within the existing literature. The number of subphases described in each model varied between five and ten. Phase number discrepancy resulted from selection of different points within the stroke cycle to denote the beginning and ending of each phase. The separation or amalgamation of subphases most commonly occurred during the propulsive and recovery phases. Further phase reductions were observed in models that did not consider pull and kick glide phases in their model.

**Table 9:** Comparison of stroke phase models utilised within the breaststroke biomechanics literature

Study	Publication year	Pull phases					Kick phases				
Chollet et al. [32]	2004	Arm glide	Arm propulsion	Elbow push	Recovery one	Recovery two	Leg propulsion	Leg insweep	Leg glide	Recovery one	Recovery two
Conceição et al. [40]	2019		First propulsive action of arms	Second propulsive actions of arms			First propulsive action of legs	Second propulsive action of legs		Recovery	
Leblanc et al. [29]	2005	Arm glide	Arm outsweep	Arm insweep	Recovery one	Recovery two	Leg propulsion	Leg insweep	Leg glide	Recovery one	Recovery two
Leblanc et al. [3]	2007		Arm propulsion		Arm and leg recovery phase		Leg propulsion			Leg-arm lag phase	
Louro et al. [34]	2016		First propulsive action of arms	Second propulsive actions of arms			First propulsive action of legs	Second propulsive action of legs		Recovery	
Nicol et al. [33]	2021		Propulsive pull		Recovery pull		Propulsive kick			Recovery kick	
Oxford et al. [14]	2017		Arm pull		Arm recovery		Leg kick			Leg recovery	
Seifert and Chollet [18]	2005	Arm glide	Arm propulsion	Elbow push	Recovery one	Recovery two	Leg propulsion	Leg insweep	Leg glide	Recovery one	Recovery two
Seifert and Chollet [36]	2009	Glide	Outsweep	Insweep	Recovery one	Recovery two	Propulsion	Insweep	Glide	Recovery one	Recovery two
Seifert et al. [37]	2014	Glide	Outsweep	Insweep	Recovery one	Recovery two	Propulsion	Insweep	Glide	Recovery one	Recovery two
Staniak et al. [28]	2016		Upper limb propulsion		Motion deceleration		Lower limb propulsion		Gliding		
Takagi et al. [2]	2004	Glide	Outsweep	Insweep	Recovery	Sweep	Lift and glide	Recovery			

Between-group differences in temporal patterns were frequently discussed within the literature. Temporal differences were associated with variations in race distance, experience level and sex. Comparison of temporal patterns between 50, 100 and 200 m race distances has identified several variations. When compared to a typical 200 m stroke, the 50 m stroke is characterised by a relative time increase in arm propulsion, arm recovery, leg propulsion and leg recovery phases [32]. Changes to the glide phase are also evident between race distances with decreases in the arm and leg glide phases common with decrease in race distance [2, 18, 32, 33]. The increase in time spent in propulsive phases and decrease in time spent in glide phases with decrease in race distance reflect a need to overcome higher drag forces at greater velocities [32]. In addition to time spent in each phase, the total distance covered during the glide phase is reported to decrease as race distance decreases from 200 to 50 m (0.50 m ± 0.25–0.22 m ± 0.20) [3]. This pattern is also true when considered relative to total stroke distance (22.14% ± 8.26–11.10% ± 5.03) [3].

At present there is no consensus on temporal variations across race duration. In a study of 26 breaststroke specialists, Oxford et al. [14] identified no temporal changes to the propulsive or recovery phases over the duration of a 100 m time trial (TT). Conversely, temporal changes have been reported to occur over the duration of a 200 m TT [34]. Temporal changes over a 200 m distance most commonly occur during the latter part of arm propulsion until the end of arm recovery and during the final 45°of leg extension [34]. Other temporal phases remain relatively stable over race duration [34]. Inconsistency in reported findings may reflect different temporal patterns between the 100 and 200 m event. This difference may otherwise be attributed to the use of different phase models between studies. Use of different models alters the calculation of time spent in each phase and makes comparison between studies difficult.

Temporal comparisons between elite and non-elite populations were also found frequently in the literature. Temporal patterns were largely consistent between elite and non-elite groups with no differences in arm propulsion, arm glide or arm recovery identified [29]. Similar findings were observed in lower limb temporal patterns. No differences in leg propulsion or leg recovery phases between elite and non-elite swimmers have been reported [29]. Despite large similarities between elite and non-elite populations, temporal differences are reported during leg recovery one phase (time between the end of leg glide and the achievement of a 90° knee angle during recovery) in 200 m pace swimming for male swimmers of different experience levels [29]. Elite males typically spend a longer amount of time in this phase when compared to non-elite males (14.20% ± 5.06 and 11.33% ± 3.36, respectively) [29]. This finding may be attributed to a proportional decrease in the leg glide phase or greater range of knee flexion during leg recovery in elite swimmers [29]. Temporal differences in female elite and non-elite populations occur during leg insweep and leg glide phases. Elite female swimmers spent longer in the leg insweep phase at 50 and 100 m paces (11.55% ± 2.09 and 9.30% ± 0.83 at 50 m pace and 11.63% ± 1.55 and 9.38% ± 0.80 at 100 m pace, respectively) and less time in the leg glide phase at 100 and 200 m paces (46.53% ± 3.55 and 53.29% ± 5.71 at 100 m pace and 49.44% ± 4.60 and 56.04% ± 6.25 at 200 m pace, respectively) [29].

Elite swimmers also typically travel further during each stroke phase when compared to non-elite swimmers [3, 26]. This is true for all temporal phases except for the glide phase at 90% of maximal speed [26]. When normalised to total stroke distance (calculated as v/SR) however, non-elite swimmers travel further than elite swimmers during leg propulsion and glide phases [3]. Elite swimmers continue to travel further during all other phases when considered in relative terms [3]. The ability of elite swimmers to travel further during each temporal phase is attributed to their ability to maintain a streamlined position with one set of limbs during the propulsive phase of the other set of limbs. This finding may also result from higher acceleration values achieved by expert swimmers throughout propulsive phases [26].

Temporal comparisons between elite populations have also been reported. In the comparison of World Championship semi-finalists and preliminary swimmers, semi-finalists typically spend longer in the arm glide phase than eliminated swimmers [2]. This pattern is consistent across all race distances and between male and female swimmers [2]. When compared to national medallist swimmers, World Championship athletes also spend less time in the leg recovery phase (0.46 s ± 0.06 and 0.37 s ± 0.09, respectively) [35]. Temporal differences observed between the highest performing athletes highlight the intricacy and complexity of temporal characteristics in elite breaststroke swimming. Further temporal investigation within elite populations is needed in order to develop a broader understanding of optimal temporal patterns.

The final characteristic for between-group temporal comparisons was sex. Male swimmers typically spend longer in propulsive phases and less time in the arm glide phase when compared to female swimmers of the same experience level and swimming intensity [18, 33]. These differences have been attributed to sex-based morphology variances. Due to their increased size and consequent large propelling surface, male swimmers can generate greater mechanical outputs than female swimmers [14]. This may explain why male swimmers spend longer in propulsive phases. The reported difference in glide time between male and female swimmers may be attributed to an increased amount of adipose tissue typical to female morphology [18]. This reduces the energy cost required to maintain a horizontal position required for an efficient glide phase [18].

Temporal characteristics were also investigated through assessment of coordination. Coordination patterns were used to assess limb synchronicity between the discontinuous propulsive phases associated with breaststroke swimming. Two methods were commonly used to describe and evaluate coordination patterns in breaststroke swimming. The first method assessed coordination patterns through measurement of a number of time gaps throughout the stroke cycle. Three time-gap models have been developed by various research groups (Table 10). The most commonly referenced time-gap model developed by Seifert and Chollet [18] assessed coordination across five time gaps [18, 36, 37]. Using this method, three modes of coordination were possible. Classification of each mode of coordination was dependent on the length of T1_*b*_ [32]. When T1_*b*_ > 0 a glide mode of coordination occurs [37]. This signifies that arm outsweep began after completion of the leg insweep phase. Opposition or continuous coordination occurs when T1_*b*_ = 0 and superposition or overlap mode occurs when T1_*b*_ < 0 [32]. This meant that the arm outsweep began at the same time, or prior to leg insweep completion [32].

**Table 10:** Comparison of time-gap models described within the breaststroke biomechanics literature

Time period	Seifert and Chollet model [18]	Oxford et al. model [14]	Takagi et al. model [2]
Time between the end of leg propulsion and beginning of arm propulsion	T1_a_	CP1	Simultaneous propulsion time
Time between the end of leg insweep and beginning of arm propulsion	T1_b_	–	–
Time between the beginning of arm recovery and the beginning of leg recovery	T2	–	–
Time between the end of arm recovery and the end of leg recovery	T3	–	–
Time between 90° arm flexion during recovery and 90° leg flexion during recovery	T4	–	–
Time between the beginning of leg propulsion and the beginning of arm propulsion	–	Arm lag time	Per cent arm lag time
Time between the end of arm propulsion and the beginning of leg propulsion	–	CP2	Simultaneous recovery time
Expression of coordination phases	% of total leg stroke time	% of total stroke time	% of total stroke time

Coordination patterns are reported to differ between race distances. Between the 200 m and 50 m event, the length of T1 decreases [32]. A similar pattern is not observed at time gaps T2, T3 and T4. These coordination points do not vary between race distances [18, 32]. The reduction in T1 indicates a shift towards a continuous or overlap mode of coordination as race distance decreases. A shift towards the continuous or overlap mode of coordination may be considered advantageous in the maintenance of a higher average velocity due to the reduction of IVV [18, 37]. These coordination modes are consequently considered most economical due to a reduction in mechanical energy output [37]. Despite a reduction in mechanical energy output, the use of continuous and overlap modes is often associated with an increase in SR and decrease in SL. The energetic cost of maintaining these modes of coordination over extended periods has not been investigated and warrants further research in order to better understand the influence of coordination mode selection on race performance.

Temporal phases and the time-gap method have been used in conjunction to assess the percentage of total stroke time spent in propulsion. Titled the index of flat breaststroke propulsion (IBFP), this parameter is calculated using a combination of leg propulsion time, arm propulsion time, elbow push time, T1_*a*_, T2 and T3 (Eqs. 4–6) [18].

If T1_*a*_ > 0

4 $${\text{IBFP}} = {\text{leg}}\;{\text{ propulsion}} + \;{\text{arm}}\;{\text{propulsion}} + \;{\text{elbow}}\;{\text{push}} - T1_{a}$$

If T1_*a*_ < 0, T2 < 0 and T3 < 0

5 $${\text{IBFP}} = {\text{leg}}\;{\text{ propulsion}} + {\text{arm}}\;{\text{ propulsion}} + {\text{elbow}}\;{\text{ push}} + \left| {\left( {T2 + T3} \right)} \right| - T1_{a}$$

If T1_*a*_ < 0, T2 > 0 and T3 > 0

6 $${\text{IBFP}} = {\text{leg}}\;{\text{ propulsion}} + {\text{arm}}\;{\text{ propulsion}} + {\text{elbow}}\;{\text{ push}}$$

Note: all phase lengths used in IBFP calculations are derived from the stroke phase model described by Seifert and Chollet [18]. The IBFP is reported to increase with decrease in race distance in female swimmers but not male swimmers [18]. Despite male swimmers maintaining a similar IBFP at all race distances, male swimmers continue to have a higher IBFP at all race distances when compared to female swimmers [18]. This finding is consistent with stroke phase analysis that has consistently found male swimmers spend longer in both arm and leg propulsive phases than female swimmers [18].

The second method of coordination pattern assessment described coordination patterns through the analysis of elbow and knee angles [26]. Titled the continuous relative phase (CRP), this method uses joint displacement and angular velocity to calculate a joint phase angle (Eq. 7) [26, 27].

7 $$\phi = \arctan \left( {\frac{{\omega_{{{\text{norm}}}} }}{{\theta_{{{\text{norm}}}} }}} \right)$$

where ${\omega }_{norm}$ refers to normalised values of angular velocity and ${\theta }_{norm}$ refers to normalised values of angular displacement. Elbow and knee phase angles are subsequently used to calculate the relative phase at a given time point (Eq. 8) [26, 27].

8 $${\text{CRP}} = \phi_{{{\text{elbow}}}} - \phi_{{{\text{knee}}}}$$

Using continuous relative phase, two modes of coordination are possible: in-phase and anti-phase. In-phase coordination occurs when − 30° < CRP < 30° and indicates that both sets of limbs are performing a similar motion (i.e. both in flexion or both in extension) [26, 27]. Anti-phase coordination occurs when − 180° < CRP < − 150° and 150° < CRP < 180° and indicates that each set of limbs is in opposing motion (i.e. one set of limbs in flexion and one in extension) [26, 27].

CRP patterns are reported to differ between individuals of various experience levels. Elite breaststroke swimmers typically exhibit lower relative phase values at maximal leg flexion when compared to recreational level swimmers [26]. This results from elite swimmers reaching maximal elbow extension earlier than recreational swimmers at the same swimming intensity [26]. Elite swimmers also exhibit lower maximal CRP values than recreational swimmers [26]. This is attributed to elite swimmers achieving full elbow and knee extension during the glide phase. Recreational swimmers in comparison maintain small amounts of elbow and knee flexion throughout this phase and consequently maintain a higher CRP [26].

**Fig. 2:**
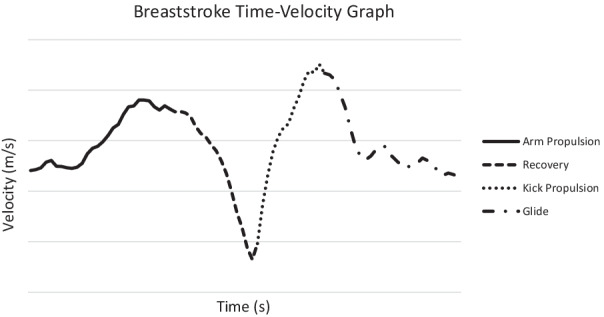
Breaststroke time–velocity chart

Using the above-described temporal models, researchers have investigated the velocity patterns associated with various stroke phases. A typical time–velocity curve of the breaststroke stroke cycle is characterised by two maximums and two minimums (Fig. 2). The time–velocity curve reaches its first minimum at maximal leg flexion. This minimum is followed by an increase and maximum in velocity that occurs with leg extension. As the legs finish extension, the time–velocity curve again decreases before the arms begin the propulsive phase. As arm propulsion is initiated the time–velocity curve reaches a second maximum before decreasing during arm and leg recovery phases [3]

Velocity maxima associated with the pull and kick propulsive phases are of similar magnitude at submaximal intensity (0.66 m/s ± 0.11 and 0.68 m/s ± 0.14, respectively) [28]. The velocity minima associated with the limb recovery and glide phases differ in magnitude at the same intensity. The velocity minimum associated with limb recovery is typically larger than that of the glide phase ( − 1.24 m/s ± 0.13 and 0.09 m/s ± 0.10, respectively) [28]

Patterns within the breaststroke velocity trace are strongly associated with race performance. Higher minimum velocity throughout the stroke cycle [2], a higher horizontal acceleration minimum during the glide phase (*r* = − 0.76), smaller maximum vertical acceleration during the leg propulsion and glide phase (*r* = 0.84) and a reduced relative time to minimum vertical acceleration during leg propulsion and glide (*r* = 0.91) are strongly associated with faster 50 m time [28]. Swimmers should consequently aim to reduce the rate of deceleration throughout the glide phase and ensure acceleration generated during the leg propulsion phase is applied along the horizontal axis in order to improve 50 m swimming time. Similar trends are yet to be established in 100 m and 200 m events and are of future research interest.

### Neuromuscular Activity

Neuromuscular activity of the triceps brachii (TB), biceps brachii (BB), trapezius (TRA), pectoralis major (PM), gastrocnemius (GAS), tibialis anterior (TA), biceps femoris (BF) and rectus femoris (RF) were most frequently reported in the literature due to their involvement in breaststroke swimming. Neuromuscular activity was discussed with reference to stroke phases, kinematics, intensity variations and experience-level-based differences.

With reference to neuromuscular activation patterns across the stroke cycle, the TB, BB and PM are reported as most active during the arm propulsive phase [13]. Activation of the GAS, TA, RF, TRA and BF is conversely highest during the leg propulsion phase [11, 13]. The stroke cycle is initiated with activation of TB. TB activation at this time results in lateral hand movement characteristic of the beginning of arm propulsion [13]. Following lateral hand movement, BB and PM are activated during the arm insweep phase in order to maximise arm propulsion [13]. The TRA is also activated during this time to assist in subsequent arm recovery [13]. Activation of TRA during arm recovery is coupled by activation of BF and GAS to initiate leg recovery [13]. These muscles remain activated until maximal knee flexion is reached. RF remains inactive throughout this phase [13]. Once maximal knee flexion is achieved, the leg propulsion phase begins. The beginning of this phase is characterised by high levels of activation in the BF, RF and TA [13]. The TA at this time is responsible for controlling ankle dorsiflexion and the positioning of the feet to promote maximal propulsion [13, 35]. Propulsion generation during this phase is aided by activation of BF and RF to enable strong knee extension [13]. During the latter part of the leg propulsion phase, GAS and TA activation increase in preparation for ankle plantarflexion required for an effective glide [13]. During this time, TRA remains active in order to maintain a streamlined position with the upper body [13].

The patterning of neuromuscular activity across the stroke cycle is relatively similar at various swimming intensities [13]. The main point of difference between various intensity bouts is the timing of TB activation. At higher intensities, TB activation occurs earlier within the stroke cycle [13]. Earlier TB activation signifies an earlier onset of the arm outsweep and a consequent reduction in glide time. This neuromuscular trend is consistent with an observed temporal shift towards continuous or overlapped modes of coordination with increasing intensity [32]. In addition to a time shift in TB activation the magnitude of neuromuscular activation changes at various swimming intensities. Increases in intensity are coupled with an increase in the sum of total integrated EMG (iEMG) [13]. This trend is consistent across TB, BB, PM, GAS, TA, BF and RF [13]. In addition to neuromuscular activity differences associated with varying intensity, neuromuscular activity is also reported to vary with changes to SR [38]. These changes are observable in the frequency of TB, BB and PM, with frequency reductions associated with an increase in breaststroke SR [38].

Several differences in neuromuscular activity patterns have been reported between beginner and elite groups. Despite some pattern similarities, activation time shifts to the PM, BB, RF and TA are common [39]. Elite swimmers typically activate TA later in the leg recovery phase when compared to beginner-level swimmers [39]. The later activation of TA during this phase delays the initiation of ankle dorsiflexion and consequently minimises drag towards the latter part of this phase [39].

Neuromuscular activity differences have also been reported within elite populations. When compared to national-elite breaststroke swimmers, international medallists typically activate BB and PM earlier in the arm propulsive phase [35]. Earlier BB and PM activation signifies an earlier onset of the arm insweep and a consequent ability to generate greater propulsion [35]. Neuromuscular differences between these two performance-level groups are also common during the leg recovery phase. International medallists typically activate BF for a longer period during this phase when compared to national-elite swimmers [35]. Longer activation indicates maintenance of more neuromuscular activity during the leg recovery phase and may explain an observed reduction in leg recovery time when compared to national-elite swimmers [35]. International medallists also typically activate TA later in this phase [35]. Similar to conclusions drawn from comparison of beginner and elite populations, the latter activation of TA at this time reduces resistive forces experienced by international medallists at maximal knee flexion [35]. Another characteristic common to international medallists is the activation of GAS during the leg glide phase [11, 35]. This suggests the use of ankle plantarflexion to reduce drag throughout the glide phase and is a characteristic not frequently seen among national-elite athletes [35]. The final point of difference between national-elite and international medallists is an increased level of TB activation during the beginning of the leg propulsion phase [35]. The higher level of TB activation observed in national-elite swimmers may indicate the onset of leg propulsion prior to the end of arm extension [35]. This observation may otherwise be indicative of the use of TB to maintain upper body streamline during the leg propulsion phase [35]. Performance-level-based comparisons in neuromuscular activity highlight a number of variances that increase propulsion, reduce resistive forces and may delay muscle fatigue onset in international medallists. Consideration of these factors should be made in the adaptation of breaststroke technique to maximise performance.

Despite emerging evidence to support neuromuscular differences between national-elite and international medallist breaststroke swimmers, the limited size of samples hinders the generalisability of reported findings to the broader elite population. Described neuromuscular differences may be partially attributed to use of individualistic neuromuscular patterns to produce the same movement [11, 40]. It is also possible that some athletes use muscles that are not frequently investigated within the literature during breaststroke swimming. Further investigation into neuromuscular activity patterns within and between elite populations is required to validate preliminary findings.

### Pacing

Few research articles have investigated breaststroke pacing profiles. Despite a small amount of research, the existing literature is in consensus regarding pacing characteristics in elite breaststroke swimming.

Elite breaststroke events consistently model a positive pacing profile. This profile is characterised by a reduction is swimming speed over each consecutive 50 m split [12, 41]. Positive pacing profiles in breaststroke swimming are commonly used across 100 and 200 m race distances, by male and female swimmers [12, 23, 42, 43] and between heat to final races [41]. The positive profile characteristic of breaststroke racing is unique within competitive swimming due to the comparatively large split time variability [41]. The reduction in speed over the duration of a race is consequently more than typical for any other event.

Despite common use of a positive pacing strategy in breaststroke swimming, debate exists regarding its effect on performance. When compared to an even pacing strategy (similar speed over consecutive 50 m splits), a positive pacing strategy is associated with high post-effort blood La^+^ and higher rate of perceived exertion [42]. These differences are attributed to a greater intensity during early stages of the effort and a consequent increase in lactate accumulation time [42]. A positive pacing strategy is also associated with higher SR over the first half of a 175 m effort when compared to an even pacing strategy [42]. SR differences are not apparent over the final half of a 175 m effort [42]. Given the increase in energy cost associated with increase in SR [31], it may be suggested that the use of a positive pacing strategy increases total energy cost over an event. In response to these findings, it has been suggested that the use of an even pacing strategy may delay the onset of fatigue [42]. The adoption of an even pacing strategy may consequently aid in the maintenance of a higher average velocity throughout an event. This hypothesis is yet to be empirically tested; however, it warrants further investigation. Also of future research interest is how the use of various pacing strategies influence biomechanical parameters including temporal patterns and propulsion characteristics.

Support for further investigation into the role of pacing profiles on overall race performance is warranted based on correlational analysis that has reported the relationship between split times and overall race time. Of all splits available, final lap time is most strongly correlated to overall time in male 100 m finalists (*r* = 0.80), female 100 m finalists (*r* = 0.83) and male 200 m finalists (*r* = 0.67) at international competition. Lap three is most strongly associated with 200 m race time in female swimmers at international competition (*r* = 0.91) [44]. Given the strong association between final lap time and overall race time, it may be expected that swimmers who are able to maintain or reserve their speed for the final lap may have a faster overall race time than swimmers who use their speed over the first lap. This racing strategy closely reflects that of an even or negative pacing strategy and may suggest the positive pacing profile most commonly adopted in breaststroke racing is not the most advantageous for minimising overall race time.

### Kinetics

An emerging area of interest in breaststroke biomechanics is kinetics. Only two of the 35 research studies included in this review considered force production in investigation of breaststroke swimming. Preliminary research into swimming force outputs has found breaststroke swimmers produce the highest absolute and relative (normalised to body mass) maximum forces of all four competitive stroke specialists [45]. This finding was attributed to the simultaneous propulsive movements of each pair of limbs and the powerful leg kick unique to breaststroke swimming [45]

Force profile characteristics have been associated with swimming and breaststroke kicking performance. Absolute maximum and average force production during a tethered 30 s maximum effort have been significantly associated with breaststroke swimming velocity at 50, 100 and 200 m distances (*r* = − 0.90, − 0.77 and − 0.66 for maximum values, respectively, and *r* = − 0.94, − 0.86 and − 0.80 for mean values, respectively) [45]. Average force production is more closely correlated to swimming velocity than maximum force production at all distances [45]. The strength of the relationship between force production and velocity decreases as race distance increases [45].

A force–velocity relationship has also been established between fluid forces acting upon the foot and breaststroke kicking performance. In an investigation of eight national-level breaststroke swimmers, Tsunokawa et al. [46] identified a strong correlation between fluid force impulse and average velocity over a 50 m breaststroke kick time trial (*r* = 0.87). The relationship between force output and kicking velocity, but was not significant when maximal force output was considered (*p* > 0.05).

Kinetics in breaststroke swimming is a relatively under-researched area of study. The strength of associations reported by Morouço et al. [45] and Tsunokawa et al. [46] should promote interest in the practical application of force testing in the elite swimming environment.

## Conclusion

Empirical investigation of elite breaststroke biomechanics over the past two decades has largely centred on kinematics, temporal analysis and neuromuscular activity. Research in these areas has typically reported between-group differences between athletes of various experience or performance level, race distance and sex. Irrespective of their prevalence within the literature, several research groups have suggested these parameters would be better investigated on an individual basis to best understand kinematics, temporal patterns and neuromuscular activity within an elite population. Research with an individualistic approach remains relatively uncommon within the relevant literature, with Sanders et al. [47] the only reviewed article to adopt a case study approach to analysis.

Despite existing shortcomings, research to date has provided coaches and performance scientists with a breadth of knowledge to influence technical prescription and optimise breaststroke swimming performance at an elite level. Based on the existing literature, coaches and performance scientists should consider the identification of an optimal SR to SL ratio on an individual basis and monitor kinematic changes across race duration. Coaches and performance scientists may also consider the temporal characteristics typical of an athlete’s primary event to ensure their athlete coordinates limb movements efficiently. With consideration of the above factors and individual athlete characteristics, coaches and performance scientists will be well positioned to make meaningful changes to breaststroke athlete performance at an elite level.

## Data Availability

Data sharing is not applicable to this article as no datasets were generated or analysed during the current study.

## Abbreviations

2D: Two-dimensions
3D: Three-dimensions
BB: Biceps brachii
BF: Biceps femoris
CP1: Coordination phase one
CP2: Coordination phase two
CRP: Continuous relative phase
EMG: Electromyography
eWPS: Effective work per stroke
FINA: Federation Internationale de Natation
GAS: Gastrocnemius
IBFP: Index of flat breaststroke propulsion
iEMG: Total integrated EMG
IVV: Intracyclic velocity variation
PM: Pectoralis major
PRISMA: Preferred reporting items for systematic reviews and meta-analyses
RF: Rectus femoris
SI: Stroke index
SL: Stroke length
SR: Stroke rate
T1_a_: Temporal gap one^a^
T1_b_: Temporal gap one^b^
T2: Temporal gap two
T3: Temporal gap three
T4: Temporal gap four
TA: Tibialis anterior
TB: Triceps brachii
TRA: Trapezius
TT: Time trial
